# Estrogen Secreted by Mesenchymal Stem Cells Necessarily Determines Their Feasibility of Therapeutical Application

**DOI:** 10.1038/srep15286

**Published:** 2015-10-19

**Authors:** Jiansha Li, Xiaochun Peng, Xianqin Zeng, Bingxun Liu, Qiang Hao, Xiangyuan Yu, Liping Zhu, Qinghua Hu

**Affiliations:** 1 Key Laboratory of Pulmonary Diseases of Ministry of Health and Department of Pathophysiology, School of Basic Medicine; 2 Department of Pathology, Tongji Hospital; Tongji Medical College, Huazhong University of Science and Technology (HUST), Wuhan 430030, People’s Republic of China

## Abstract

Mesenchymal stem cells are therapeutically applicable and involved in the development of some types of diseases including estrogen (E2)-related ones. Little is known about E2 secretion by mesenchymal stem cells and its potential influence on their therapeutical applications. Our *in vitro* experiments showed that BMSCs cultured from C57BL/6J mice secreted E2 in a time-dependent manner. *In vivo* study identified a significantly increased E2 level in serum after a single administration of BMSCs, and a sustained elevation of E2 level upon a repetitive administration. Morris water maze test in the ovariectomised (OVX) mouse model revealed BMSCs transplantation ameliorated OVX-induced memory deficits by secreted E2. On the contrary, in endometriosis model, BMSCs transplantation aggravated endometriotic lesions because of E2 secretion. Mechanistically, the aromatase cytochrome P450 appeared to be critical for the biosynthesis and exerted effects of estrogen secretion by BMSCs. Our findings suggested that BMSCs transplantation is on the one hand an attractive option for the therapeutic treatment of diseases associated with E2 deficits in part through E2 secretion, on the other hand a detrimental factor for the E2-exasperated diseases largely via E2 production. It is important and necessary to monitor serum E2 level before and after the initiation of BMSCs therapy.

The transplantation of mesenchymal stem cells is a valuable treatment against many diseases. Mesenchymal stem cells are also implicated in the development of some types of diseases including estrogen (E2) related disorders^1^,^2^,^3^. However, little is known about E2 secretion from mesenchymal stem cells and the potential influence of E2 on their therapeutical application.

E2 has been reported to possess memory-enhancing effects and therefore become a treatment option for memory impairment^4^,^5^. But, there is always debate about hormone therapies including E2 agents since they increase the risk of stroke and dementia^6^,^7^. Several clinical reports revealed that bone marrow-derived mesenchymal stem cells (BMSCs) improved the functional recovery of stroke patients without adverse side effects^8^,^9^. The latest laboratory study found that female BMSCs cultured in high glucose medium differentiated into steroidogenic cells with the ability to synthesize and release E2^10^. This implied the possibility and advantage of BMSCs in cell therapy against memory impairment.

On the contrary, with the potential ability to produce E2, BMSCs therapy may bring great disadvantages to the host who suffers from high E2-worsen diseases. For example, endometriosis is an estrogen-dependent gynecological disease where endometrium-like tissue grows outside uterine cavity. BMSCs transplantation may aggravate the endometriotic lesion^3^,^11^.

Facing these contradictions, we tested in this study the status of E2 secretion from mouse BMSCs, and evaluated its potential impact on the application of BMSCs for two distinct disease models, which are highly associated with E2 deficiency or E2 excess respectively.

## Materials and Methods

### Ethics statement and the animals used

The procedures for all animal experiments were approved by the Institutional Animal Care and Use Committee of the Tongji Medical College, and carried out in accordance with the National Institutes of Health Guide for the Care and Use of Laboratory Animals. C57BL/6J mice and SD rats (3 to 4-weeks old) were used for bone marrow-derived mesenchymal stem cell isolation^12^,^13^,^14^. Female C57BL/6J mice (6 to 8-weeks old) were used for stem cell transplantation as well as ovariectomised (OVX) models and endometriosis models.

### Primary culture and identification of BMSCs

BMSCs obtained from the bone marrow of female C57BL/6J mice and SD rat by flushing the femur and tibia diaphyses with Dulbecco’s modified Eagle’s medium (DMEM) were cultured in DMEM supplemented with 20% fetal bovine serum (FBS), 100 U/ml penicillin and 100 mg/ml streptomycin. The cultured cells were identified as BMSCs by Fluorescence Activated Cell Sorting (FACS) as previously described^12^,^13^. Briefly, cultured BMSCs at passage 3 were adjusted to the density of 2 × l0^6^/ml after being trypsinized with 0.25% trypsin, and then incubated with fluorescence-conjugated antibodies for CD29, CD34, CD45 as well as their isotype controls in a black chamber at 4 °C, respectively; 30 min later, cells analysis was performed with a flow cytometry (Becton Dickenson, USA) after washing with PBS. BMSCs at passage 3 were used for the following experiments.

### Western blot assay

Whole cell protein samples were prepared by lysing the primarily cultured female and male BMSCs at passage 3. Equal amounts of protein extracts were western-blotted for the following specific antibodies: rabbit polyclonal antibodies to aromatase cytochrome P450 (CYP19A1, Proteintech, USA), steroid sulfatase (Proteintech, USA) and 17β-HSD (Boster Biological Technology CO, China). Mouse monoclonal antibody β-actin served as control for equal protein loading.

### E2 measurements *in vitro* experimental protocol

BMSCs were re-incubated into 6-well culture plates at a density of 2 × 10^5^/well and the culture medium was collected at 0, 1, 2, 3 and 4 days after culture separately for determining the levels of E2 by radioimmunoassay (RIA) in clinical laboratory of Tongji Hospital affiliated to Tongji Medical College (Wuhan, China). Meanwhile, the remnant cells in each well were digested and counted for further use of E2 concentration calibration.

### E2 measurements *in vivo* experimental protocol

C57BL/6J mice were sacrificed with blood withdrawal by retrobulbor venous puncture under light ether anesthesia with or without the administration of BMSCs via tail vein (2 × 10^6^ cells in 0.1 ml PBS)^12^. The blood samples were collected from the mice at 24 and 72 hours after they received a single injection of BMSCs, and also collected at 96 hours from the mice receiving a single injection of BMSCs and from the mice receiving an additional/repeated injection at 72 hours after the first injection of BMSCs. 1 ml collected blood was laid up for 2 h at room temperature, followed by centrifugation at 3000 rpm for 10 minutes. The obtained serum was collected for measurement of E2 concentration by RIA. The blood samples were also collected for measurement of serum E2 level at the 6 th day from the mice after they received all the five injections of BMSCs in Morris water maze studies, or at the 2 nd day from the mice after they received all the five injections of BMSCs in endometriosis studies as described below in details, respectively.

### Plasmid and stable transfection

BMSCs from C57BL/6J mice were seeded at density of 2 × 10^5^/well into a 6-well plate in DMEM supplemented with 10% FBS. BMSCs undergoing exponential growth were concurrently transfected with 2 μg P450 empty (pGCsilencerTM U6/Neo/GFP/RNAi vector from the Genechem Corporation, Shanghai, China) or P450 siRNA (pGCsilencerTM U6/Neo/GFP/RNAi-P450 from the Genechem Corporation, Shanghai, China) for the control group or siRNA group, respectively. Transfection was performed using the Lipofectamine 2000 reagent (Invitrogen, CA, USA) according to the manufacturer’s recommendations. At 48 hours after transfection, the cells were trypsinized and plated for clonal selection of stable transfectants in 400 μg/ml G-418. With observation under immunofluorescence microscope, about 70% clones with good expression of the genes were selected out of 48 individual clones in each group.

### Morris water maze test

C57BL/6J mice were bilaterally ovariectomized (OVX) following standardized procedures^15^. Then, they received subcutaneous injections of E2 (0.02 mg E2/100 mg mouse body weight) or BMSCs transplantation every three days for half a month. Subsequently, the standard Morris water maze procedure was used with minor modifications^16^,^17^,^18^. Briefly, mice were trained to find a hidden platform in water maze for 6 consecutive days, four trials per day with a 30-s interval. The mice were not allowed to search the platform for more than 60 s, after which they were guided to the platform and stayed on the platform for 30 s. In each trial, the swimming pathway and latency locating the hidden platform was recorded using Noldus video tracking system (Ethovision, Noldus Information Technology, Holland). On each day, the mice were released from the first quadrant, and the time duration from the beginning of the release to finding of the central platform was calculated and recorded as escape time. In this study, the learning ability was quantified as escape time on the 6th day^17^,^18^. The shorter escape time a mouse needed to find the central platform on 6th day, the better it scored its spatial memory. After the experiment, all mice were sacrificed and serum was collected for measure of E2 level.

### Endometriosis models

Endometriosis mice models were produced by surgery and endometrium autotransplantation^19^. Briefly, under aseptic precautions, mice in estrus were anesthetized with ketamine (0.1 mg ketamine/kg mouse body weight, i.p.). After exposure of the uterus by midline abdominal incision, a segment sized about 1 cm of the left uterine horn was removed. Then, from this segment, two pieces of uterine horn (~2 mm × 2 mm) were cut and sewn into bilateral abdominal wall. Subsequently, they were received E2 injection or BMSCs transplantation every three days. Half a month later, all mice were sacrificed; the cystic endometriotic lesions were harvested from the bilateral abdominal wall and then the sizes were measured. Meanwhile, the serum was collected for measurement of E2 level.

### Statistical analysis

The data were expressed as the mean ± SEM and analyzed using SPSS 12.0 statistical software (SPSS Inc., Chicago, Illinois, USA). One-way analysis of variance followed by least significant difference post hoc analysis was used to determine the statistical significance of the differences between the means.

## Results

### E2 secretion by BMSCs

**Figure 1:**
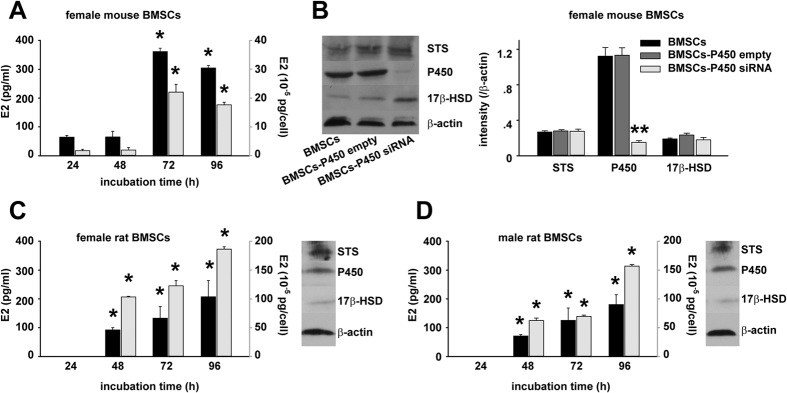
E2 secretion by mouse and rat BMSCs. (**A**) Supernatant of cultured female mouse BMSCs were collected separately at 24, 48, 72 and 96 h after incubation. The E2 levels (Y-axis on the left) determined by RIA were also calibrated per cell (Y-axis on the right) (**B**) Protein expression levels for P450, STS and 17β-HSD in female mouse BMSCs with/without P450 siRNA plasmid transfection were evaluated by western blot assay and quantitative analysis, β-actin served as a control for equal protein loading. Band densities of P450, STS and 17β-HSD were normalized by β-actin. (**C**,**D**) Supernatant of female (**C**) and male rat BMSCs (**D**) were collected separately at 24, 48, 72 and 96 h after incubation. The E2 levels (Y-axis on the left) determined by RIA were also calibrated per cell (Y-axis on the right). Protein expression of P450, STS and 17β-HSD in female and male rat BMSCs were detected by western blots. *p < 0.05 versus 24 h, **p < 0.01 versus BMSC or BMSCs-P450 empty group. n = 3 separate experiments for each.

To evaluate E2 secretion from cultured BMSCs, the culture medium of mouse and rat BMSCs were collected and the E2 levels were measured by RIA. To eliminate the disturbance of background value, we first detected E2 levels in cultured pure culture medium at 24, 48, and 72 h separately. They were all at about 90 pg/ml. Therefore, 90 pg/ml was considered the background value and was deducted from the following data detected for the culture medium of mouse and rat BMSCs. At 24 h after incubation with female mouse BMSCs, there was a low concentration of E2 in the culture medium. Then the E2 level jumped to a peak level at 72 h, then slightly fell down and maintained at a high level for at least an additional 24 h (Fig. 1A). To be more accurate, the E2 levels in the culture medium were normalized by the number of BMSCs in each well. The calibrated E2 level per BMSC increased in the same time-dependent manner, as shown in Fig. 1A. While in rat BMSCs, there was no detectable E2 secretion in either female or male cells at 24 h after incubation. In the following durations from 24 h to 96 h, the level of E2 concentration in culture medium as well as calibrated E2 level per BMSC, both increased in a similar time-dependent manner (Fig. 1C,D).

To elucidate E2 production capability of BMSCs, we examined protein expression about the important enzymes involved in estrogen biosynthesis, including aromatase cytochrome P450 (P450), steroid sulfatase (STS) and 17β-hydroxysteroid dehydrogenases (17β-HSD)^20^,^21^. Western blots revealed the protein expression of P450, STS and 17β-HSD in BMSCs (Fig. 1B–D). Since there was always a higher expression of P450 than the other two enzymes (Fig. 1B–D), we further constructed specific siRNA plasmid against P450 and confirmed its efficiency by the western blot assay (Fig. 1B). Collectively, the above data strongly demonstrated E2 secretion capability of BMSCs.

### BMSCs transplantation changed E2 level of serum in C57BL/6J mice by E2 secretion

**Figure 2:**
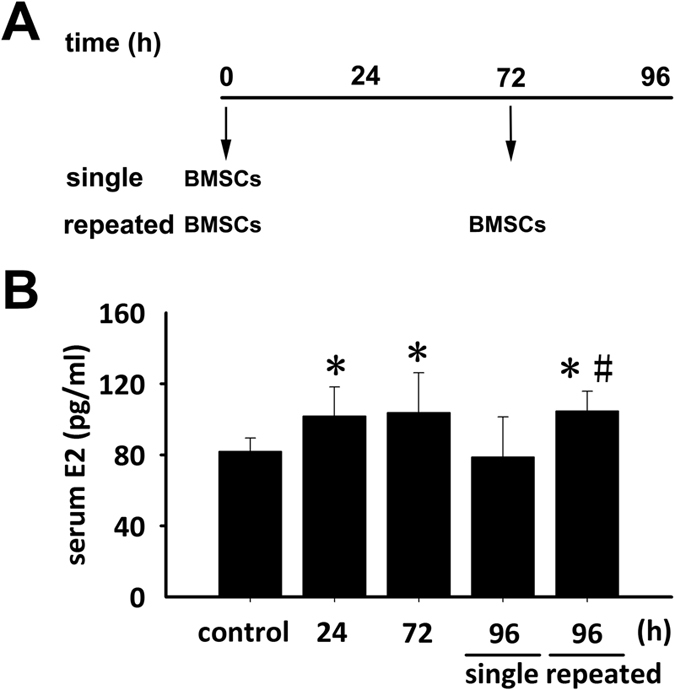
Serum E2 level in recipient mice after BMSCs transplantation. (**A**) The schematic diagram showing C57BL/6J mice in separate groups treated by a single injection of BMSCs followed with or without a repeated injection at 72 h after the first one, and blood samples from treated mice and control mice without any injection of BMSCs were collected for serum E2 measurements. (**B**) Serum E2 levels in recipient mice and control counterparts (*P < 0.05 versus control mice without any injection of BMSCs, ^**#**^*P* < 0.05 versus mice with a single injection of BMSCs, n = 6 for each group).

In this study, C57BL/6J mice were separately treated by a single injection of BMSCs followed with or without a repeated injection at 72 h after the first one (Fig. 2A), and serum from treated mice and control mice without any injection of BMSCs were collected for E2 measurements. After a single administration of BMSCs, E2 level in the mouse serum significantly increased at 24 h, maintained for an additional 48 h and then fell down to a level close to the baseline at 96 h (Fig. 2B). Upon a repeated administration of BMSCs at 72 h, E2 level in the mouse serum maintained at the high level throughout the observation period (^*^P < 0.05 vs. mice without injection of BMSCs, ^**#**^*P* < 0.05 vs. mice with a single injection of BMSCs, Fig. 2B).

### BMSCs transplantation ameliorated OVX-induced memory deficits in mice

**Figure 3:**
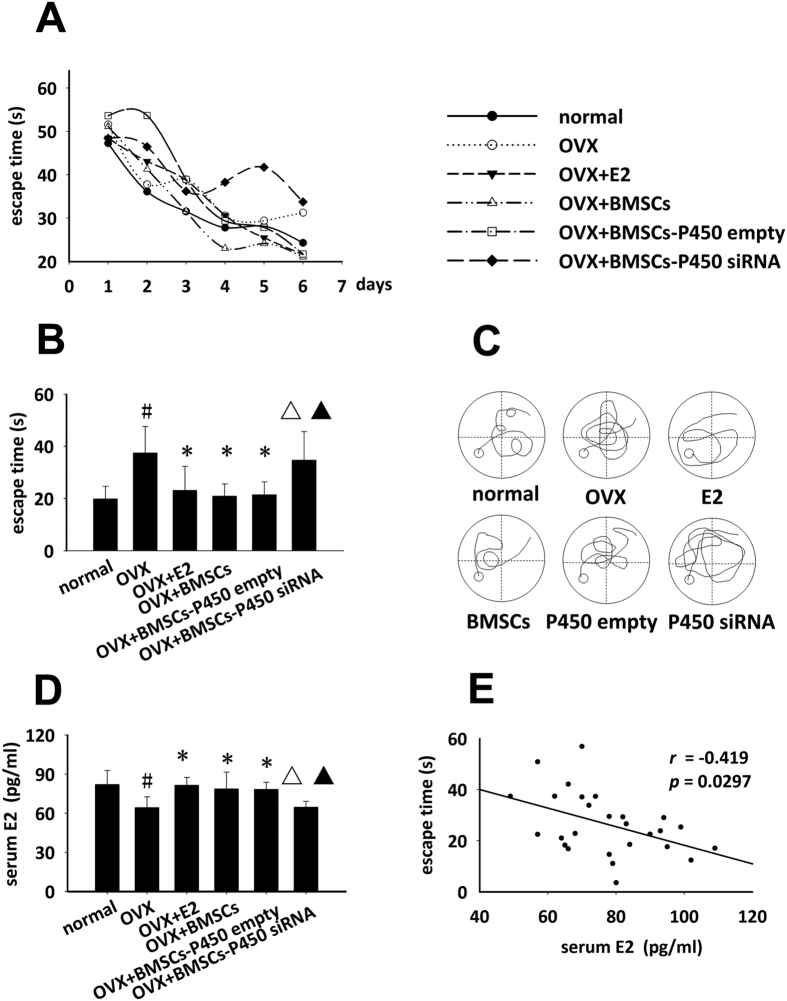
BMSCs transplantation ameliorated OVX-induced memory deficits in mice via E2 secretion. After OVX, the mice were treated with E2 injection or BMSCs transplantation with/without manipulation of P450 expressions for half a month, followed by a consecutive training for 6 days with the Morris water maze. (**A**) The averaged escape time on every day for each group to find the hidden platform, (**B**) Statistical analysis of escape time on the 6th day to find the hidden platform, (**C**) The representative path to find the platform on the 6th day, (**D**) Serum E2 level detected by collecting serum of all the mice after Morris water maze test, (**E**) The regressive analysis of serum E2 levels and the escape time. ^#^*p* < 0.05 versus normal group, *p < 0.05 versus OVX, ^△^*p* < 0.05 versus OVX+BMSCs, ^▲^*p* < 0.05 versus OVX+BMSCs-P450 empty, n = 11 for each.

Since BMSCs transplantation was able to increase serum E2 level in C57BL/6J mice, we then investigated whether BMSCs transplantation would affect memory deficits induced by OVX. After OVX, the mice were received treatment for half a month by E2 injection or BMSCs transplantation with/without manipulation of protein P450, the product of the CYP19 gene, which is one of the most important enzymes responsible for estrogen biosynthesis^20^,^21^. Then the learning ability of mice was examined with the Morris water maze. During the 6 days of consecutive training in the Morris water maze, the averaged escape time on each day were shown in Fig. 3A, and the escape time on the 6th day for the mice to find the hidden platform was used to quantify their learning ability (Fig. 3A,B)^17^,^18^. As shown in Fig. 3B, compared with the normal group, the escape time significantly increased in mice receiving OVX (^#^*P* < 0.05, Fig. 3B), implying the memory deficit models were successfully produced. Similarly and slightly more efficient than E2 injection (the improvement of escape time by E2 administration was ~86% by BSMCs), BMSCs transplantation reversed the memory impairment induced by OVX (^*^P < 0.05 versus OVX group, Fig. 3B), however, siRNA knockdown of P450 in BMSCs largely abolished their improvement on learning ability (Fig. 3A–C). Consistently, compared with OVX group, the serum E2 levels were elevated in OVX+E2 group or in OVX+BMSCs group for at least six days after all administrations (^*^P < 0.05), however not changed in those with P450-knockdown BMSCs transplantation (Fig. 3D). The data indicated that BMSCs transplantation possessed slightly greater function than E2 injection in reversing memory impairment associated with OVX. Furthermore, As shown in Fig. 3B,D, there were also significant differences about the escape time as well as serum E2 level between OVX+BMSCs group and OVX+BMSC-P450 siRNA group (^△^*P* < 0.05), and also between BMSCs-P450 siRNA group and BMSCs-P450 empty group (^▲^*P* < 0.05), suggesting E2 production ability of BMSCs through the activity of P450 and its importance in BMSCs treatment against memory deficits. The regression analysis revealed a negative relationship between the E2 levels and the learning ability (Fig. 3E). Taken together, BMSCs transplantation ameliorated OVX-induced memory deficits in part through P450-regulated E2 secretion.

### BMSCs transplantation aggravated endometriosis by E2 secretion

**Figure 4:**
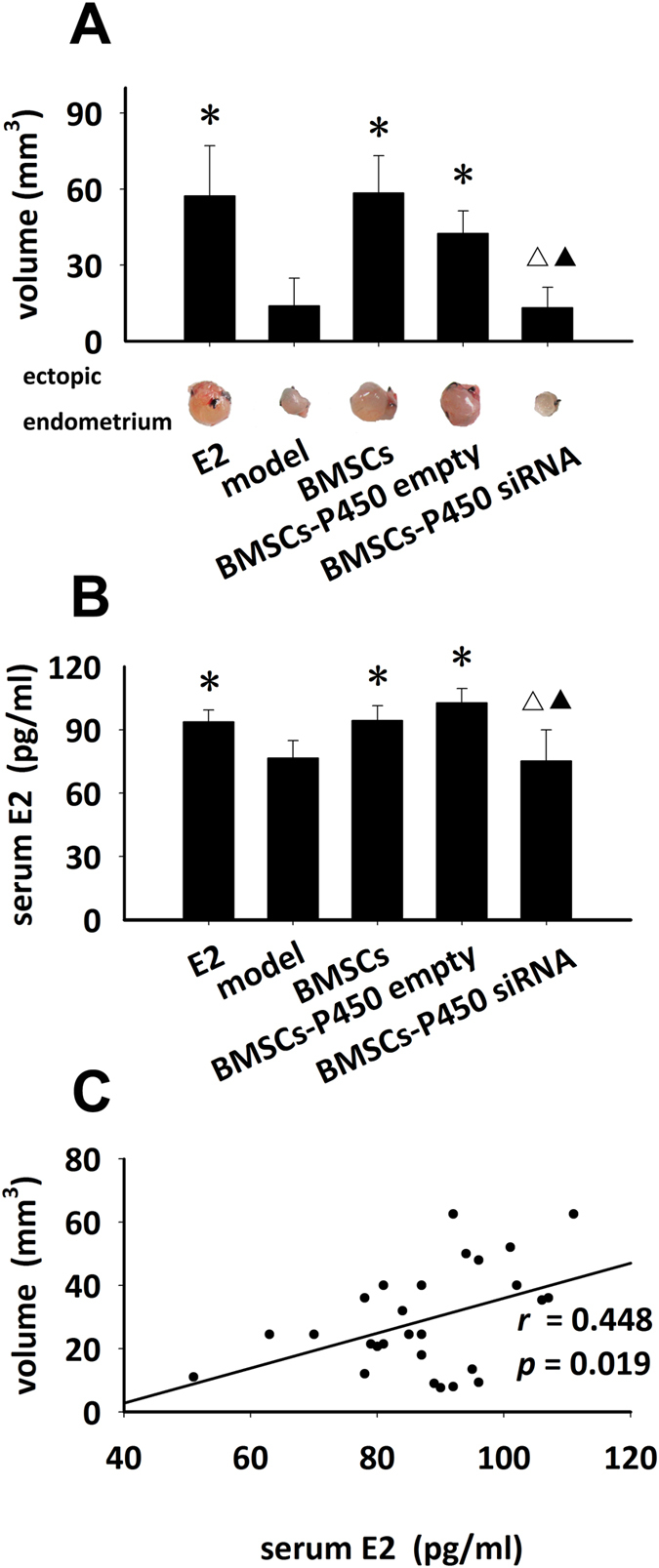
BMSCs transplantation promoted ectopic endometrium in endometriosis model. After endometriotic lesions formed, the mice were treated with E2 injection or BMSCs transplantation with/without manipulation of P450 expressions for half a month. (**A**) The representative gross view and quantitative analysis of endometriotic lesions, (**B**) The serum E2 level, (**C**) The regressive analysis of serum E2 levels and the volume of endometriotic lesions. *p < 0.05 versus model, ^△^*p* < 0.05 versus BMSCs, ^▲^*p* < 0.05 versus BMSCs-P450 empty, n = 11 for each.

Finally, experiments were designed to evaluate whether BMSCs transplantation would also affect endometriosis. After endometriosis was surgically induced, the mice were treated by E2 injection or BMSCs transplantation with/without manipulation of P450 protein for half a month. As shown in Fig. 4A, similar as in E2 group, the volume of the endometriotic lesions was significantly larger in the mice receiving BMSCs transplantation than that in model group (^*^P < 0.05 versus model group), however, no apparent difference was found in the volume of the endometriotic lesions between BMSCs-P450 siRNA group and model group. Together with the evidences about the significant difference between BMSCs group and BMSCs-P450 siRNA group (^△^*P *< 0.05), as well as between BMSCs-P450 siRNA and BMSCs-P450 empty group (^▲^*P* < 0.05), the data highly suggested that the negative effects of BMSCs transplantation on endometriosis was most possibly via E2 secretion. As expected, the following detection of the serum E2 levels in all the mice showed similar change tendency among the above groups and further supported our speculation (Fig. 4B). Statistically, there was a positive relationship between E2 level and the volume of the endometriotic lesions (Fig. 4C). These results demonstrated that BMSCs transplantation aggravated endometriosis possibly through E2 secretion.

## Discussion

In recent years, BMSCs transplantation has been broadly recognized as a promising strategy for many diseases, including estrogen-deficiency-associated ones such as memory deficits^1^,^2^. However, few studies ever examined the status of E2 secretion by BMSCs. The latest study showed that BMSCs from SD rats cultured under a high glucose condition were able to differenciate into steroidogenic cells and then secrete E2^10^. To the best of our knowledge, in previous studies, few efforts were made to analyze the effect of BMSCs transplantation on the E2 level of animals. Furthermore, there has never been a study aimed at determining the possible influence of E2 secretion from BMSCs on their eligibilities of therapeutic application. In the present study, we found that E2 level in the culture medium of rat BMSCs increased in a time-dependent manner (Fig. 1). Our study suggested that E2 secretion from BMSCs is a common phenomenon since it was also present in the culture medium of mouse BMSCs. The presence of protein expression of 3 important enzymes for E2 biosynthesis including P450, STS and 17β-HSD in BMSCs strengthened our finding (Fig. 1). Furthermore, BMSCs retained their abilities to secret E2 after transplantation *in vivo* and more importantly the secreted E2 significantly affected the serum E2 level in the mice receiving BMSCs administration.

It has been reported that repeated administration resulted in better therapeutic effects versus a single injection of BMSCs against diseases such as liver failure^22^ and chronic kidney disease^23^. Lee SR *et al.* employed weekly administration of BMSCs^23^. Here, based on the data about changes of E2 levels with time in the culture medium of BMSCs, which showed a slight decline beginning from 72 h, in experiments *in vivo*, we gave a repeated BMSCs injection every 3 days, which was proven to be a feasible method to make serum E2 level of model mice maintain a high level. Accordingly, we purposely compared the effect of a single versus the repeated administrations of BMSCs on E2 serum levels. We found that the repeated administration induced sustained elevation of E2 levels (Figs 2, 3 and 4) and even a single one was still capable of inducing a transient elevation (Fig. 2).

An important aim of this present study was to know whether the ability of BMSCs in E2 secretion influences their therapeutical application. Recently, several studies demonstrated that E2 at high serum level enhanced learning and memory^24^. It is well known in recent decades that there is a positive correlation between high levels of E2 and hippocampal memory^25^. Using OVX-mediated memory deficits mouse, a typical disease model directly related with E2 deficiency^26^,^27^, we found that repeated BMSCs transplantation, similar as E2 treatment, was of considerable therapeutical efficacy. Furthermore, by knocking down protein expression of P450, an important enzyme in E2 biosynthesis^20^,^21^, BMSCs greatly lost the therapeutic function against memory deficits, implied a critical role of E2 secretion in their therapeutic efficacy. However, the data in our study showed a tendency about better function of BMSCs transplantation than E2 injection, suggesting besides E2 secretion, some other mechanisms might be involved in BMSCs function against memory impairment. In some former studies reported by other labs, the explanations about therapeutic function of BMSCs against memory deficits were mainly around a variety of cytokines and growth factors which were involved in neurorestorative or inflammatory processes^28^,^29^,^30^,^31^,^32^. Taken together, in the current study, we cannot exclude the other mechanisms; however, E2 secretion in part contributed to the therapeutic function of BMSCs in E2-deficiency associated disease such as OVX-induced memory deficits.

Since BMSCs possess the ability of E2 secretion, another fact we need to be aware of is that high E2 level is also a major pathogenic and or contributing factor for development of some diseases, such as endometriosis^33^,^34^. Endometriosis is a common estrogen-dependent gynecological disease characterized by growth of endometrial tissue outside the uterine cavity. It is believed increased E2 levels stimulate proliferation of endometrium. To address any potential adverse effects of BMSCs due to their E2 secretion, we employed endometriosis mouse model in the present study. Our data verified that the volume of the endometriotic lesions became significantly larger in mice with transplantation of BMSCs and the changed degree is very close to that in the ones with E2 treatment, while they showed no change after transplantation of P450-knockdown BMSCs (Fig. 4). P450 is well known to be a key enzyme in the production of E2^20^,^21^. Therefore, our data strongly suggested that this disadvantage of BMSCs administration in endometriosis model was largely due to E2 secretion. A few previous studies demonstrated the contribution of BMSCs in development of endometriosis^35^,^36^. However, the explanations were mainly around the ability of BMSCs to differentiate into epithelial cells^35^ and repopulation of endometrium^36^. They further implied, during the above process, that E2 might play a key role in stimulating BMSCs’ epithelial differentiation in the process of endometriosis^35^. Here, according to data presented in Fig. 4, we raised an alternative explanation: BMSCs contribute to the development of endometriosis mainly via E2 secretion.

In clinic, E2 level is highly associated with many diseases. For example, it is well known decreased E2 serum level may contribute to development of Alzheimer disease^26^, cardiovascular diseases^37^, and osteoporotic fracture^38^. On the other hand, increased E2 serum level may result in endometriosis^33^,^34^, endometrial carcinoma^39^, obesity and breast cancer^40^. In the current study, by *in vitro* and *in vivo* experiments, we found BMSCs possessed the ability of E2 secretion and BMSCs transplantation was able to increase the serum level of E2. Therefore, the administration of BMSCs can be a double-edged sword, a possible panacea for the diseases associated with E2 deficiency, but a possible catastrophe for the diseases resulted from high E2 level. The ability of E2 secretion should be taken into consideration when employing BMSCs for treatment.

## Conclusion

We here for the first time indicated that transplantation of mouse BMSCs increased serum E2 level by E2 secretion in C57BL/6J mice, and therefore ameliorated OVX-induced memory deficits but aggravated endometriosis. The ability of E2 secretion of BMSCS at least partially determines their eligibilities of therapeutic application.

## Additional Information

**How to cite this article**: Li, J. *et al.* Estrogen Secreted by Mesenchymal Stem Cells Necessarily Determines Their Feasibility of Therapeutical Application. *Sci. Rep.* **5**, 15286; doi: 10.1038/srep15286 (2015).
